# Correlation between metformin use and mortality in acute respiratory failure: a retrospective ICU cohort study

**DOI:** 10.3389/fphar.2025.1584230

**Published:** 2025-08-26

**Authors:** Yunlin Yang, Jinfeng Liu, Yi Hou, Yuxun Wei, Liang Huang, Wei Wei

**Affiliations:** ^1^ Department of Clinical Pharmacy, Shifang People’s Hospital, Shifang, Sichuan, China; ^2^ Department of Pharmacy, People’s Hospital of Zhongjiang County, Deyang, China; ^3^ Department of Pharmacy and Evidence-Based Pharmacy Center, West China Second University Hospital, Chengdu, China; ^4^ Key Laboratory of Birth Defects and Related Diseases of Women and Children (Sichuan University), Ministry of Education, Chengdu, Sichuan, China

**Keywords:** intensive care unit, acute respiratory failure, retrospective cohort study, mortality, metformin

## Abstract

**Background:**

The aim of this study was to investigate the association of metformin use with the risk of in-hospital mortality and prognosis in acute respiratory failure (ARF) patients admitted to the intensive care unit (ICU).

**Methods:**

We conducted a retrospective cohort study using the MIMIC-IV database. Patients were categorized into metformin and non-metformin groups based on medication exposure. Primary outcomes were in-hospital and ICU mortality, while 30-day and 90-day all-cause mortality served as secondary endpoints. We applied Kaplan–Meier survival curves, Cox proportional hazards models, and logistic regression to assess associations. Propensity score matching (PSM) and machine learning algorithms were used for confounder adjustment and feature selection.

**Results:**

After PSM, 1,429 patients with ARF were included (374 metformin users; 1,055 non-users). Multivariate logistic regression revealed that metformin use was associated with significantly reduced in-hospital mortality (OR = 0.202, 95% CI: 0.123–0.317, p < 0.001) and ICU mortality (OR = 0.245, 95% CI: 0.142–0.399, p < 0.001). Cox models showed consistent reductions in 30-day (HR = 0.199, 95% CI: 0.124–0.320, p < 0.001) and 90-day (HR = 0.230, 95% CI: 0.150–0.352, p < 0.001) mortality. Kaplan–Meier curves confirmed better survival in the metformin group (p < 0.001). Subgroup analyses supported a consistent protective effect of metformin across most patient strata.

**Conclusion:**

Metformin use was significantly associated with decreased short-term mortality among ICU patients with ARF. These findings suggest that metformin, beyond its glucose-lowering effects, may offer survival benefits in critically ill populations. Clinicians should consider the potential role of metformin when managing ICU patients with type 2 diabetes and ARF. Further prospective studies are warranted to confirm these findings and optimize clinical application strategies.

## Introduction

Acute respiratory failure (ARF) is defined as acute and progressive hypoxemia in previously healthy patients due to a variety of cardiopulmonary or systemic diseases (Fujishima, 2023), and it is one of the most common and life-threatening complications among critically ill patients. A recent binational ICU study reported that over 50% of patients developed acute hypoxaemic respiratory failure within 24 h, with in-hospital mortality ranging from 8% in mild to 30% in severe cases (Ling et al., 2024). The pathogenesis of ARF is complex and may involve a variety of pathological processes such as immune dysregulation, inflammatory responses, oxidative stress, and metabolic disturbances (Lu et al., 2022; Wang et al., 2022). However, current treatments for ARF focus on supportive therapies and lack specific pharmacologic interventions (Fujishima et al., 2020).

Metformin is a classical drug widely used in the treatment of type 2 diabetes, which has the effects of lowering blood glucose and improving insulin sensitivity, while its anti-inflammatory, antioxidant, and immunomodulatory effects have received increasing attention in recent years (LaMoia and Shulman, 2021; Rena, Hardie, and Pearson, 2017). Studies have shown that metformin can inhibit the release of inflammatory factors, reduce oxidative stress, and protect organ function by activating the AMP-activated protein kinase (AMPK) signaling pathway (T. Liu et al., 2023; Bai and Chen, 2021; Lin et al., 2023). These properties make metformin an important candidate with therapeutic potential for critically ill patients.

Metformin treatment has been reported to be associated with reduced mortality from lower respiratory disease in diabetic patient (Mendy et al., 2019). Moreover, there is growing evidence that metformin reduces the production of pro-inflammatory factors *in vitro* and mitigates inflammatory harm *in vivo* (Kim and Choi, 2012; Yuan et al., 2012; Liu et al., 2017; Koh et al., 2014), and reduces all-cause mortality in patients with sepsis (Liang et al., 2019). However, there is still a lack of studies related to the use of metformin in patients with ARF and its impact on prognosis.

The primary objective of this study was to evaluate the association between metformin use and in-hospital and ICU mortality in critically ill patients with ARF. Secondary objectives included assessing 30-day and 90-day mortality following ICU admission, aiming to elucidate the potential impact of metformin on clinical outcomes in this patient population.

## Methods

### Study population and data source

This is a retrospective observational study. The Medical Information Mart for Intensive Care-IV (MIMIC-IV-3.0) is a publicly available database including more than 94,000 intensive care unit (ICU) admissions from the Beth Israel Deaconess Medical Center, spanning the years 2008–2022 (Johnson et al., 2023). The MIMIC-IV database has extensive information, including demographic data, vital signs, laboratory findings, and diagnoses. It is classified with the International Classification of Diseases, Ninth Edition (ICD-9) and Tenth Edition (ICD-10) codes. To access this database, one author (WW) obtained the necessary certification and subsequently extracted the relevant variables for our study (certification number: 13149633).

**FIGURE 1:**
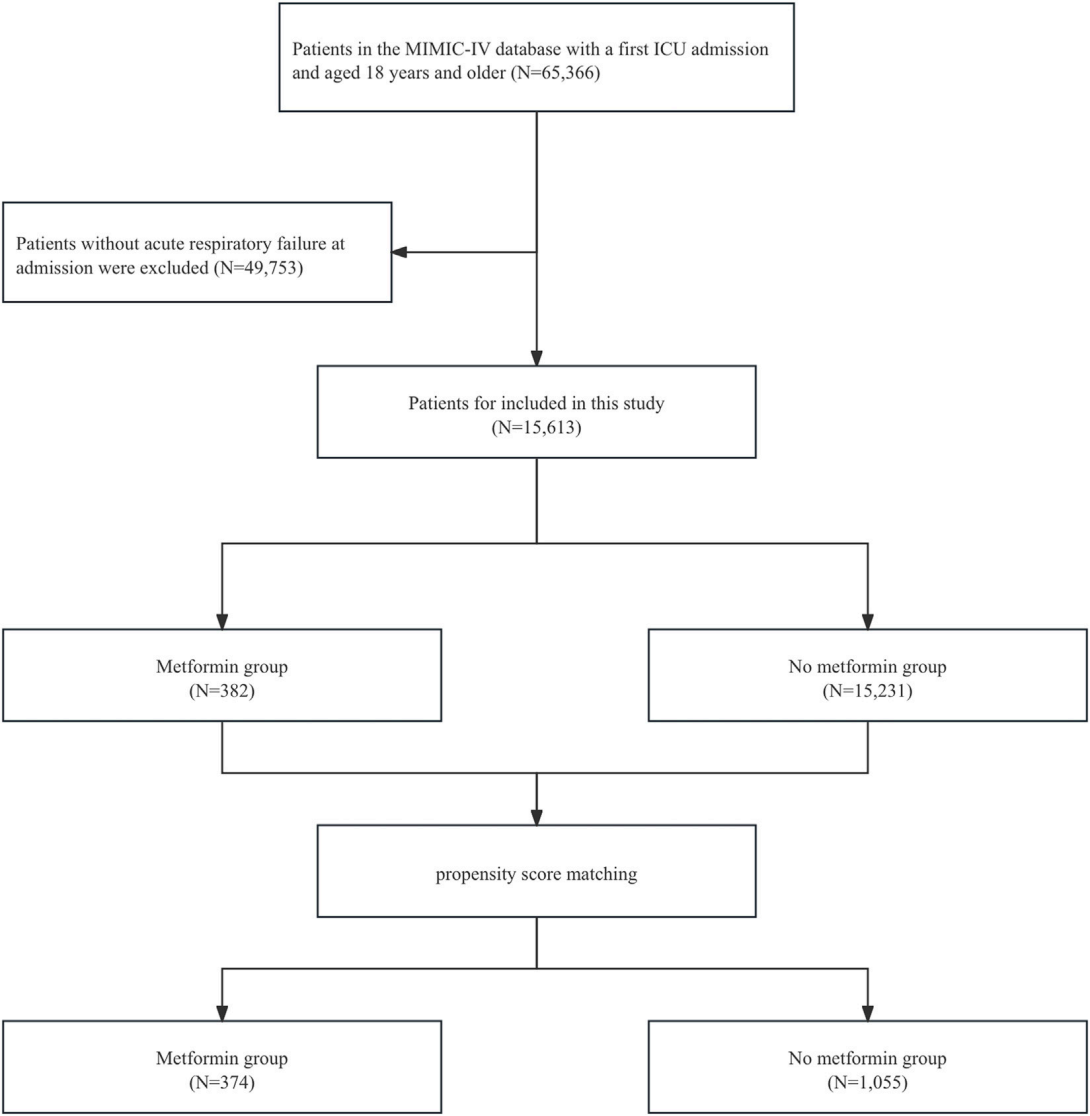
Flowchart of this study. Abbreviations: MIMIC, Medical Information Mart for Intensive Care; ICU, intensive care unit.

We included patients diagnosed with ARF upon admission in the MIMIC-IV database. The codes employed for data extraction were International Classification of Diseases ninth edition diagnostic codes (“51,851”, “51,881”) and 10th edition diagnostic codes (“J95821”, “J960”, “J9601”, “J9602”). The final enrollment of 15,613 patients with a diagnosis of ARF was divided into a metformin group (382 patients) and a non-metformin group (15,231 patients) (Figure 1). Patients were defined as metformin users if they received metformin at any point during their ICU stay, based on medication administration records in the MIMIC-IV database. Those without any record of ICU metformin use were classified as non-users. The follow-up period started at ICU admission, with endpoints defined according to the outcome measures. Patients were followed from ICU admission until hospital discharge or death. For secondary outcomes (30-day and 90-day mortality), follow-up ended at either the specified time point or death, whichever occurred first.

### Data extraction

Baseline characteristics were retrieved from the MIMIC-IV database using Structured Query Language (SQL) in combination with PostgreSQL (version 9.6), encompassing age, gender, and severity at admission SOFA score, APSIII, SIRS score, and SAPSII, as well as comorbidities and laboratory variables recorded within 24 h of ICU admission. Coronary heart disease (CHD), heart failure, hypertension, myocardial infarction, hyperlipidemia (HLD), diabetes, acute kidney injury (AKI), chronic kidney disease (CKD), cancer (CA), chronic obstructive pulmonary disease (COPD), liver cirrhosis (LC), hepatitis (HEP), pneumonia (PNA), cerebrovascular accident (CVA), chronic bronchitis (CB), and ischemic heart disease (IHD) were defined with ICD-9 and ICD-10 codes.

### Primary outcomes and secondary outcomes

The primary outcomes of this study were hospitalized all-cause mortality and ICU mortality, and the secondary outcomes were 30-day mortality and 90-day mortality after ICU admission.

### Selection of features

Before examining the relationship between metformin administration and in-hospital mortality in patients with ARF, we first utilized machine learning methods for feature selection to determine their relevance in prognostic models. An essential technique in this context is the Boruta algorithm, a prevalent approach for feature selection. The algorithm’s core is founded on two concepts: “shaded features” and “binomial distributions.” Boruta creates a collection of feature replicas from the original dataset, referred to as shaded features. If a feature’s Z-score above the maximum Z-score of a shaded feature, it is deemed significant and retained; if not, it is discarded (Degenhardt et al., 2019). In addition, we used the Random Forest model for variable feature selection and the Shapley Additive Extension (SHAP) package to visualize variable importance (Lundberg et al., 2020). The SHAP package, implemented through the SHAP Python package (version 0.39.0), facilitates model interpretation to mitigate the inherent black-box nature of machine learning, thereby helping clinicians understand the results provided by the model (Nohara et al., 2022).

### Statistical analysis

Continuous variables were summarized as mean ± standard deviation or median (IQR) and compared using Student’s t-test or Mann–Whitney U test where appropriate. Categorical variables were summarized as frequencies and percentages, with group differences evaluated using the Pearson chi-square test or Fisher’s exact test. Missing data were handled using multiple imputations, and variables with >30% missingness were excluded.

Propensity score matching (1:3 nearest neighbor with a caliper of 0.2) was used to reduce baseline differences, and balance between groups was assessed using standardized mean difference (SMD), with an SMD >0.1 considered meaningful (Austin, 2011). To further address potential residual imbalance and improve precision, multivariable regression was conducted on the matched cohort (Ali et al., 2019).

**FIGURE 2:**
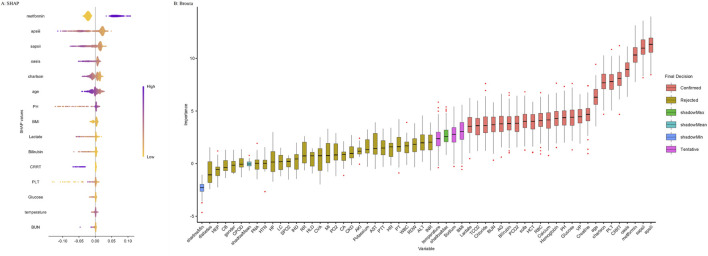
Application of Machine Learning in Feature Selection. **(A)** Shapley Additive Explanations (SHAP) for the random forest model. **(A)** Distribution of the impact of each feature on the model output. Each dot represents a patient in a row. The colors of the dots represent the feature values: purple represents larger values and yellow represents lower values. **(B)** Feature selection for the relationship between various variables and in-hospital mortality was analyzed using the Boruta algorithm. **(B)** The horizontal axis shows the name of each variable, whereas the vertical axis represents the Z-value of each variable. The box plot depicts the Z-value of each variable in the model calculation, with red boxes representing important variables, pink boxes representing tentative attributes, and yellow boxes representing unimportant variables. Abbreviations: APSIII, acute physiology score III; SAPSII, simplified acute physiology score II; OASIS, Oxford acute severity of illness score; CRRT, continuous renal replacement therapy; PLT, platelets; Charlson, Charlson comorbidity index; VP, vasopressor; PH, potential of hydrogen; RBC, red blood cell; HCT, hematocrit; SOFA, sequential organ failure assessment; PCO2, partial pressure of carbon dioxide; AG, anion gap; BUN, blood urea nitrogen; TCO2, total carbon dioxide; BMI, body mass index; INR, international normalized ratio; ALT, alanine aminotransferase; RDW, red cell distribution width; WBC, white blood cell; PT, prothrombin time; HR, heart rate; PTT, partial thromboplastin time; AST, aspartate aminotransferase; AKI, acute kidney injury; CKD, chronic kidney disease; CA, cancer; PO2, partial pressure of oxygen; MI, myocardial infarction; CVA, cerebrovascular accident; HLD, hyperlipidemia; RR, respiratory rate; IHD, ischemic heart disease; SPO2, peripheral capillary oxygen saturation; LC, liver cirrhosis; HF, heart failure; HTN, hypertension; PNA, pneumonia; COPD, chronic obstructive pulmonary disease; CB, chronic bronchitis; HEP, hepatitis.

Multivariable logistic regression was conducted to assess the association between metformin use and in-hospital or ICU mortality. Three models were developed: Model 1 (unadjusted), Model 2 (adjusted for age, gender, and BMI), and Model 3 (adjusted for covariates selected via Boruta and random forest feature selection). The importance of variables selected via Boruta and random forest algorithms is illustrated in Figure 2, which visualizes the ranking of features contributing to model construction. Cox proportional hazards regression was used to evaluate the association with 30- and 90-day all-cause mortality. Additionally, subgroup analyses were conducted to investigate the relationship between metformin use and 30-day as well as 90-day mortality across various subgroups. To address potential immortal time bias, we performed landmark analyses at two time points—48 h and 7 days after ICU admission (Suissa, 2007). For each landmark, only patients who survived beyond the respective time point were included. Patients were classified as metformin users if they received metformin within the landmark window (i.e., within 48 h or within 7 days of ICU admission). Those who had not received metformin prior to the landmark time point were classified as non-users. Kaplan–Meier survival curves were generated to compare 30-day and 90-day mortality between groups for each landmark time point. To improve comparability, both unmatched and PSM cohorts were used in the landmark analyses, and group differences were assessed using log-rank tests. All statistical analyses were performed using R software (version 4.1.2, R Foundation). A two-sided P-value below 0.05 was considered statistically significant. This study followed the STROBE guidelines for reporting observational studies (von Elm et al., 2007).

## Results

### Baseline characteristics

This study included 15,613 patients who met the eligibility criteria, with 15,231 in the non-metformin group and 382 in the metformin group. Our study is a retrospective study, and its results are susceptible to confounding factors. After PSM, 1,429 patients were included, and confounding was minimized. Adequate balance was achieved across most baseline characteristics (Supplementary Figure S1). Table 1 presents the clinical data of the two patient groups prior to PSM. In the original population, there was no difference in age between the metformin group and the non-metformin group, while for clinical scores such as assessed via the Sequential Organ Failure Assessment (SOFA), Oxford acute severity of illness score (OASIS), Acute Physiology Score III (APSIII), and Simplified Acute Physiology Score II (SAPSII), the metformin group was lower than the non-metformin group. In addition, for patient clinical outcomes, patients in the metformin group had lower ICU mortality and in-hospital mortality compared with patients in the non-metformin group. After PSM, there were no significant differences in baseline characteristics between the two groups (Table 2).

**TABLE 1:** Baseline characteristics of the original population.

Categories	Overall (N = 15,613)	Metformin group (N = 382)	No metformin group(N = 15,231)	*P*-value	SMD
Age, years	68.00 (57.00–79.00)	66.00 (58.00–76.00)	69.00 (57.00–80.00)	0.37	0.05
Male, n (%)	8,700 (55.72%)	223 (58.38%)	8,477 (55.66%)	0.31	0.05
BMI, kg/m^2^	27.59 (23.60–32.56)	31.22 (25.77–36.91)	27.51 (23.56–32.46)	<0.01	0.39
SOFA score	6.00 (3.00–9.00)	4.00 (2.00–6.00)	6.00 (3.00–9.00)	<0.01	0.46
OASIS	35.00 (29.00–41.00)	31.50 (27.00–38.00)	35.00 (29.00–41.00)	<0.01	0.32
APSIII	48.00 (36.00–66.00)	41.00 (31.25–53.00)	49.00 (36.00–66.00)	<0.01	0.39
SAPSII	40.00 (31.00–51.00)	36.00 (27.00–44.00)	40.00 (31.00–51.00)	<0.01	0.35
CHARLSON	6.00 (3.00–8.00)	5.00 (4.00–7.00)	6.00 (3.00–8.00)	0.10	0.09
Comorbidities, n (%)					
Heart failure	5,627 (36.04)	120 (31.41)	5,507 (36.16)	0.06	0.10
Hypertension	5,669 (36.31)	233 (60.99)	5,436 (35.69)	<0.01	0.52
Myocardial infarction	1,609 (56.89)	39 (10.21)	1,570 (10.31)	1.00	<0.01
HLD	5,627 (36.04)	222 (58.12)	5,405 (35.49)	<0.01	0.47
Diabetes	4,953 (31.72)	360 (94.24)	4,953 (30.16)	<0.01	1.76
AKI	7,788 (49.88)	144 (37.70)	7,644 (50.19)	<0.01	0.25
CKD	3,544 (22.70)	39 (10.21)	3,505 (23.01)	<0.01	0.35
CA	2,627 (16.83)	50 (13.09)	2,577 (16.92)	0.06	0.11
COPD	3,168 (20.29)	71 (18.59)	3,097 (20.33)	0.44	0.04
LC	1,422 (9.11)	10 (2.62)	1,412 (9.27)	<0.01	0.28
HEP	875 (5.60)	10 (2.62)	865 (5.68)	0.01	0.15
PNA	7,382 (47.28)	140 (36.65)	7,242 (47.55)	<0.01	0.22
CVA	1,418 (9.08)	35 (9.16)	1,383 (9.08)	1.00	<0.01
CB	1994 (12.77)	45 (11.78)	1949 (12.80)	0.61	0.03
IHD	5,398 (34.57)	166 (43.46)	5,232 (34.35)	<0.01	0.19
CRRT	1,420 (10.57)	12 (3.14)	1,408 (9.24)	<0.01	0.26
VP	9,311 (59.64)	226 (59.16)	9,085 (59.65)	0.89	0.01
Laboratory tests					
WBC, K/ul	11.60 (8.00–16.30)	12.20 (8.80–16.10)	11.50 (8.00–16.30)	0.63	0.03
RBC, m/ul	3.53 (2.98–4.12)	3.76 (3.17–4.23)	3.53 (2.98–4.11)	<0.01	0.21
RDW, %	14.90 (13.70–16.70)	14.30 (13.33–15.30)	14.90 (13.70–16.70)	<0.01	0.39
HCT, %	32.50 (27.60–37.40)	33.20 (28.50–37.40)	32.40 (27.50–37.40)	0.13	0.08
Platelet, K/ul	195.00 (135.00–265.00)	210.00 (166.25–270.75)	195.00 (157.00–259.50)	0.01	0.16
Glucose, mg/dL	134.00 (107.00–177.00)	176.00 (134.00–233.50)	133.00 (107.00–175.00)	<0.01	0.44
Hemoglobin, g/dL	10.50 (8.80–12.20)	10.80 (9.12–12.30)	10.50 (8.80–12.20)	0.06	0.10
Lactate, mmol/L	1.70 (1.20–2.80)	1.80 (1.20–2.70)	1.70 (1.20–2.80)	0.08	0.10
Serum potassium, mEq/L	4.20 (3.80–4.70)	4.20 (3.82–4.70)	4.20 (3.80–4.70)	0.94	<0.01
Serum sodium, mEq/L	139.00 (135.00–142.00)	138.00 (135.00–141.00)	139.00 (135.00–142.00)	0.14	0.08
Serum calcium, mg/dL	8.30 (7.80–8.80)	8.40 (7.90–8.90)	8.30 (7.80–8.80)	0.04	0.12
ALT, IU/L	27.00 (15.00–59.00)	26.00 (15.00–46.75)	27.00 (15.00–60.00)	0.06	0.12
AST, IU/L	39.00 (23.00–90.00)	33.00 (21.00–63.75)	40.00 (23.00–90.00)	0.03	0.14
Bilirubin, mg/dL	0.60 (0.40–1.10)	0.50 (0.30–0.78)	0.60 (0.40–1.10)	<0.01	0.33
AG, mEq/L	15.00 (12.00–18.00)	14.00 (11.00–16.00)	15.00 (12.00–18.00)	<0.01	0.25
TCO2, mEq/L	24.00 (21.00–28.00)	25.00 (22.00–28.00)	24.00 (21.00–28.00)	0.14	0.08
PCO2, mm Hg	42.00 (36.00–50.00)	43.00 (37.00–49.00)	42.00 (36.00–50.00)	0.72	0.02
PO2, mm Hg	83.00 (50.00–150.00)	105.00 (63.00–198.50)	83.00 (50.00–149.00)	<0.01	0.27
Creatine, mg/dL	1.10 (0.80–1.80)	0.90 (0.70–1.20)	1.10 (0.80–1.80)	<0.01	0.46
PT, sec	14.40 (12.60–18.00)	13.90 (12.50–16.00)	14.40 (12.60–18.10)	<0.01	0.22
PTT, sec	31.30 (27.50–38.80)	29.60 (26.60–34.75)	31.40 (27.50–38.90)	0.03	0.11
INR	1.30 (1.10–1.60)	1.30 (1.10–1.50)	1.30 (1.10–1.70)	<0.01	0.23
BUN, mg/dL	23.00 (15.00–38.00)	18.00 (14.00–25.00)	23.00 (15.00–39.00)	<0.01	0.51
Chloride, mEq/L	103.00 (99.00–108.00)	103.50 (99.00–107.00)	103.00 (99.00–108.00)	0.92	0.01
Vital signs					
RR, insp/min	20 (16–25)	20 (16–24)	20 (16–25)	0.10	0.09
HR, insp/min	91 (77–107)	90 (77–104)	91 (77–107)	0.73	0.02
Temperature, °F	98.20 (97.70–98.90)	98.20 (97.70–98.90)	98.20 (97.70–98.90)	0.04	0.14
Events					
ICU mortality, n (%)	4,088 (26.18)	20 (5.24)	4,068 (26.71)	<0.01	0.61
Hospital mortality, n (%)	4,983 (31.92)	24 (6.28)	4,959 (32.56)	<0.01	0.7

Abbreviations: SMD, standardized mean difference; BMI, body mass index; SOFA, sequential organ failure assessment; OASIS, oxford acute severity of illness score; APSIII, acute physiology score III; SAPSII, simplified acute physiology score II; HLD, hyperlipidemia; AKI, acute kidney injury; CKD, chronic kidney disease; CA, cancer; COPD, chronic obstructive pulmonary disease; LC, liver cirrhosis; HEP, hepatitis; PNA, pneumonia; CVA, cerebrovascular accident; CB, chronic bronchitis; IHD, ischemic heart disease; CRRT, continuous renal replacement therapy; VP, vasopressor; WBC, white blood cell; RBC, red blood cell; RDW, red cell distribution width; HCT, hematocrit; ALT, alanine aminotransferase; AST, aspartate aminotransferase; AG, anion gap; TCO2, total carbon dioxide; PCO2, partial pressure of carbon dioxide; PO2, partial pressure of oxygen; PT, prothrombin time; PTT, partial thromboplastin time; INR, international normalized ratio; BUN, blood urea nitrogen; RR, respiratory rate; HR, heart rate; ICU, intensive care unit.

**TABLE 2:** Characteristics of the study population after propensity score matching.

Categories	Overall (N = 1,429)	Metformin group (N = 374)	No metformin group (N = 1,055)	*P*-value	SMD
Age, years	67.00 (57.00–77.00)	66.00 (58.00–76.00)	67.00 (57.00–77.00)	0.77	0.02
Male, n (%)	812 (56.82)	216 (57.75)	596 (56.49)	0.72	0.03
BMI, kg/m^2^	30.61 (25.48–36.20)	31.64 (25.68–36.72)	30.24 (25.44–36.00)	0.92	0.01
SOFA score	4.00 (2.00–7.00)	4.00 (2.00–6.00)	4.00 (2.00–7.00)	0.71	0.02
OASIS	33.00 (28.00–39.00)	32.00 (27.00–38.00)	33.00 (28.00–39.00)	0.28	0.07
APSIII	42.00 (32.00–55.00)	41.00 (32.00–53.00)	43.00 (32.00–56.00)	0.24	0.07
SAPSII	36.00 (27.00–44.00)	36.00 (27.00–44.00)	36.00 (27.00–45.00)	0.49	0.04
CHARLSON	5.00 (4.00–7.00)	5.00 (4.00–7.00)	5.00 (4.00–7.00)	0.56	0.04
Comorbidities, n (%)					
Heart failure	468 (32.75)	120 (32.09)	348 (32.99)	0.80	0.02
Hypertension	843 (58.99)	225 (60.16)	618 (58.58)	0.64	0.03
Myocardial infarction	143 (10.01)	38 (10.16)	105 (9.95)	0.99	0.01
HLD	785 (54.93)	214 (57.22)	571 (54.12)	0.33	0.06
Diabetes	1,334 (93.35)	352 (94.12)	982 (93.08)	0.57	0.04
AKI	563 (39.40)	143 (38.24)	420 (39.81)	0.64	0.03
CKD	159 (11.13)	39 (10.43)	120 (11.37)	0.69	0.03
CA	179 (12.53)	50 (13.37)	129 (12.23)	0.63	0.03
COPD	270 (18.89)	71 (18.98)	199 (18.86)	1.00	<0.01
LC	52 (3.64)	10 (2.67)	42 (3.98)	0.32	0.07
HEP	44 (3.08)	10 (2.67)	34 (3.22)	0.72	0.03
PNA	552 (38.63)	140 (37.43)	412 (39.05)	0.62	0.03
CVA	147 (10.29)	34 (9.09)	113 (10.71)	0.43	0.05
CB	183 (12.81)	45 (12.03)	138 (13.08)	0.67	0.03
IHD	577 (40.38)	158 (42.25)	419 (39.72)	0.43	0.05
CRRT	49 (3.43)	12 (3.21)	37 (3.51)	0.91	0.02
VP	856 (59.90)	218 (58.29)	638 (60.47)	0.50	0.04
Laboratory tests					
WBC, K/ul	11.70 (8.50–15.90)	12.10 (8.70–16.10)	11.60 (8.40–15.75)	0.36	0.05
RBC, m/ul	3.74 (3.19–4.22)	3.78 (3.17–4.26)	3.72 (3.20–4.21)	0.74	0.02
RDW, %	14.30 (13.40–15.50)	14.30 (13.40–15.30)	14.30 (13.40–15.60)	0.69	0.02
HCT, %	33.50 (28.60–38.10)	33.40 (28.50–37.50)	33.70 (28.65–38.20)	0.56	0.04
Platelet, K/ul	210.00 (159.00–273.00)	211.50 (167.00–273.25)	209.00 (157.00–272.50)	0.74	0.02
Glucose, mg/dL	176.00 (130.00–237.00)	176.00 (134.00–233.50)	176.00 (130.00–238.00)	0.72	0.02
Hemoglobin, g/dL	10.90 (9.30–12.40)	10.85 (9.12–12.30)	10.90 (9.30–12.50)	0.55	0.04
Lactate, mmol/L	1.70 (1.20–2.70)	1.80 (1.20–2.60)	1.70 (1.20–2.70)	0.88	0.01
Serum potassium, mEq/L	4.20 (3.80–4.60)	4.20 (3.80–4.70)	4.20 (3.80–4.60)	0.63	0.03
Serum sodium, mEq/L	138.00 (135.00–141.00)	138.00 (135.00–141.00)	138.00 (135.00–141.00)	0.77	0.02
Serum calcium, mg/dL	8.40 (7.90–8.90)	8.40 (7.90–8.90)	8.40 (7.90–8.80)	0.30	0.06
ALT, IU/L	25.00 (15.00–47.00)	25.00 (16.00–44.00)	25.00 (15.00–47.00)	0.88	0.01
AST, IU/L	35.00 (22.00–67.00)	33.00 (21.00–62.75)	36.00 (22.00–68.00)	0.76	0.02
Bilirubin, mg/dL	0.50 (0.30–0.80)	0.50 (0.30–0.80)	0.50 (0.30–0.80)	0.82	0.01
AG, mEq/L	14.00 (11.00–16.00)	14.00 (11.00–16.00)	14.00 (12.00–16.00)	0.59	0.03
TCO2, mEq/L	25.00 (22.00–29.00)	25.00 (22.00–28.75)	25.00 (22.00–29.00)	0.81	0.02
PCO2, mm Hg	43.00 (37.00–51.00)	43.00 (38.00–50.00)	42.00 (36.00–51.00)	0.69	0.02
PO2, mm Hg	98.00 (58.00–186.00)	100.50 (60.25–189.50)	98.00 (57.00–182.50)	0.69	0.02
Creatine, mg/dL	0.90 (0.70–1.20)	0.90 (0.70–1.20)	0.90 (0.70–1.20)	0.85	0.01
PT, sec	13.70 (12.30–16.00)	13.70 (12.40–15.78)	13.60 (12.30–16.00)	0.80	0.02
PTT, sec	29.90 (26.70–34.90)	29.25 (26.60–34.80)	30.10 (26.75–35.20)	0.75	0.02
INR	1.20 (1.10–1.50)	1.20 (1.10–1.40)	1.20 (1.10–1.50)	0.72	0.02
BUN, mg/dL	18.00 (14.00–25.00)	18.00 (14.00–25.00)	19.00 (14.00–26.00)	0.86	0.01
Chloride, mEq/L	103.00 (99.00–107.00)	103.00 (99.00–107.00)	103.00 (99.00–107.00)	0.95	<0.01
Vital signs					
RR, insp/min	19 (16–24)	20 (16–24)	19 (16–24)	0.18	0.08
HR, insp/min	90 (77–104)	90 (78–105)	90 (77–104)	0.41	0.05
Temperature, °F	98.30 (97.80–99.00)	98.40 (97.80–98.97)	98.30 (97.80–99.00)	0.92	0.01
Events					
ICU mortality, n (%)	205 (14.35)	19 (5.08)	186 (17.63)	<0.01	0.40
Hospital mortality, n (%)	267 (18.68)	23 (6.15)	244 (23.13)	<0.01	0.49

Abbreviations: SMD, standardized mean difference; BMI, body mass index; SOFA, sequential organ failure assessment; OASIS, oxford acute severity of illness score; APSIII, acute physiology score III; SAPSII, simplified acute physiology score II; HLD, hyperlipidemia; AKI, acute kidney injury; CKD, chronic kidney disease; CA, cancer; COPD, chronic obstructive pulmonary disease; LC, liver cirrhosis; HEP, hepatitis; PNA, pneumonia; CVA, cerebrovascular accident; CB, chronic bronchitis; IHD, ischemic heart disease; CRRT, continuous renal replacement therapy; VP, vasopressor; WBC, white blood cell; RBC, red blood cell; RDW, red cell distribution width; HCT, hematocrit; ALT, alanine aminotransferase; AST, aspartate aminotransferase; AG, anion gap; TCO2, total carbon dioxide; PCO2, partial pressure of carbon dioxide; PO2, partial pressure of oxygen; PT, prothrombin time; PTT, partial thromboplastin time; INR, international normalized ratio; BUN, blood urea nitrogen; RR, respiratory rate; HR, heart rate; ICU, intensive care unit.

### Metformin use and hospitalization, ICU mortality rates

Compared with patients who did not use metformin, the unadjusted model showed a 78.2% reduction in the risk of in-hospital death (OR = 0.218, 95% CI 0.136–0.333, p < 0.001) and a 75.0% reduction in the risk of ICU death in patients who used metformin (OR = 0.250, 95% CI 0.149–0.397, p < 0.001). After adjusting for confounders, the protective effect of metformin was consistent across multifactorial analyses of in-hospital and ICU deaths. Fully adjusted models showed a 79.8% reduction in the risk of in-hospital death (OR = 0.202, 95% CI 0.123–0.317, p < 0.001) and a 75.5% reduction in the risk of ICU death in patients using metformin (OR = 0.245, 95% CI 0.142–0.399, p < 0.001). These findings are summarized in Table 3, which presents the logistic regression results for in-hospital and ICU mortality.

**TABLE 3:** The association between metformin and in-hospital and ICU mortality in patients with ARF.

Outcomes	Model 1		Model 2		Model 3	
	Or (95% CI)	*p*-value	Or (95% CI)	*p*-value	Or (95% CI)	*p*-value
In-hospital mortality	OR = 0.218 (0.136–0.333)	*p* < 0.001	OR = 0.215 (0.134–0.330)	*p* < 0.001	OR = 0.202 (0.123–0.317)	*p* < 0.001
ICU-mortality	OR = 0.250 (0.149–0.397)	*p* < 0.001	OR = 0.249 (0.148–0.397)	*p* < 0.001	OR = 0.245 (0.142–0.399)	*p* < 0.001

Model 1: unadjusted.

Model 2: adjusted for age; gender; BMI.

Model 3: adjusted for age; gender; BMI; APSIII; SAPSII; metformin; OASIS; CRRT; PLT; CHARLSON; creatine; VP; glucose; PH; hemoglobin; calcium; RBC; HCT; SOFA; PCO2; bilirubin; AG; BUN; chloride; TCO2; lactate.

These results suggest that metformin use significantly reduces in-hospital and ICU mortality in patients with ARF, supporting its potentially important clinical protective role in patients with ARF.

### Survival analysis and COX proportional risk modeling

**FIGURE 3:**
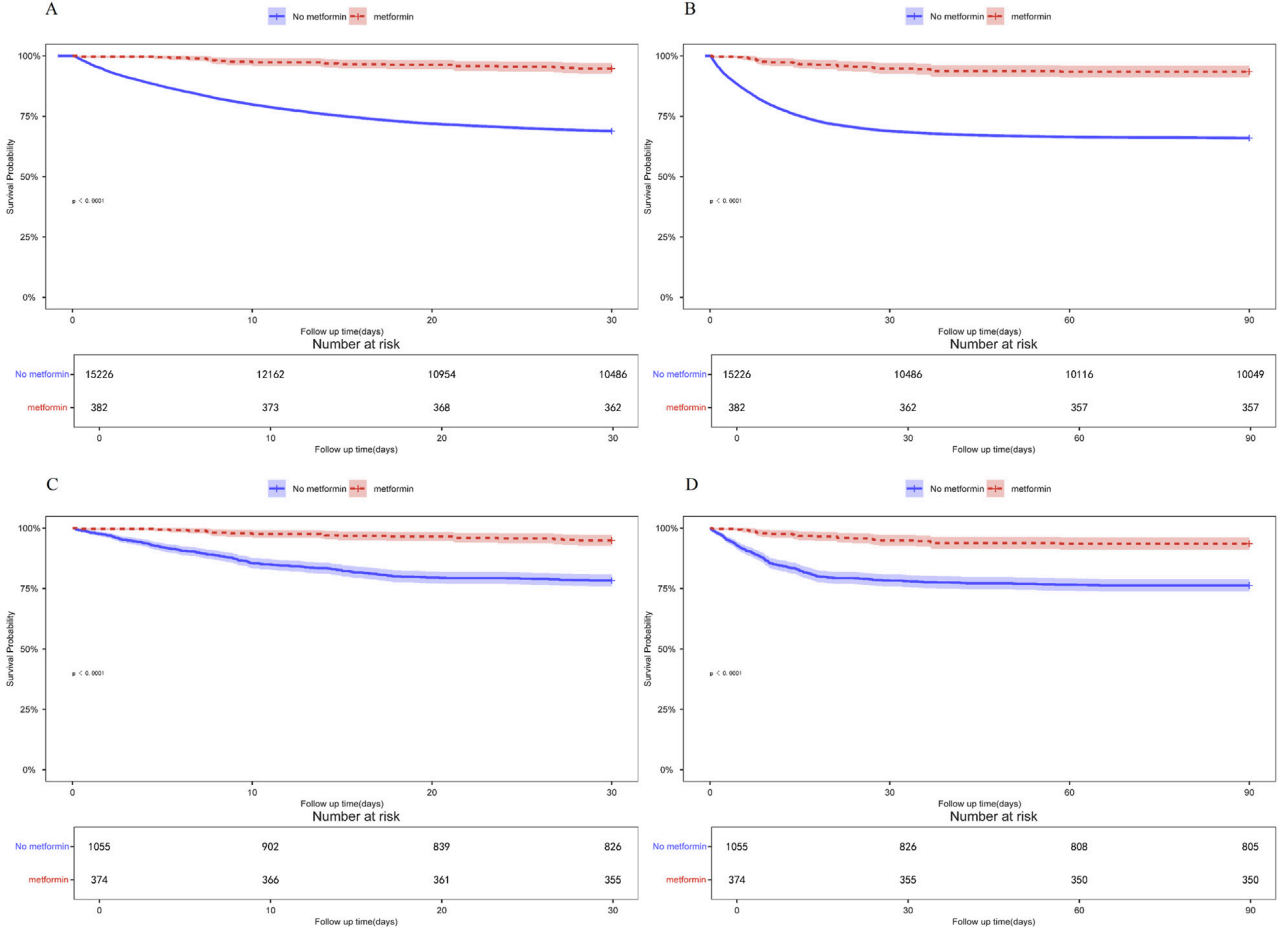
**(A)** Kaplan-Meier curves of 30-day mortality risk in the two groups for the original population. **(B)** Kaplan-Meier curves of 90-day mortality risk in the two groups for the original population. **(C)** Kaplan Meier curve of 30-day mortality risk in two groups for the PSM population. **(D)** Kaplan Meier curve of 90-day mortality risk in two groups for the PSM population.

Patients with ARF were categorized into two groups based on their metformin use during hospitalization. Univariate COX risk analysis showed that the 30-day, 90-day risk of death was lower in the metformin group than in the non-metformin group (HR = 0.211, 95% CI 0.132–0.337, p < 0.001; HR = 0.232, 95% CI 0.159–0.367, p < 0.001) (Table 4). Kaplan-Meier curves for 30- and 90-day survival are shown in Figure 3. According to Kaplan-Meier survival analysis, the 30-day survival and 90-day survival rates were significantly higher in the metformin group than in the non-metformin group (the log-rank test: p-value <0.001). We examined the association between metformin and mortality in ARF patients through Cox regression analysis, along with other factors that may affect survival. The metformin group exhibited a reduced 30-day mortality risk and a diminished 90-day mortality risk compared to the non-metformin group (adjusted HR = 0.199, 95% CI: 0.124, 0.320; adjusted HR = 0.230, 95% CI: 0.150, 0.352) as determined by multivariable Cox proportional hazards analysis (Table 4).

**TABLE 4:** Values of HR and 95% CI of metformin use for 30-day and 90-day mortality.

Outcomes	Model 1		Model 2		Model 3	
	HR (95% CI)	*p*-value	HR (95% CI)	p-value	HR (95% CI)	p-value
30-day mortality	HR = 0.211 (0.132–0.337)	*p* < 0.001	HR = 0.209 (0.131–0.334)	p < 0.001	HR = 0.199 (0.124–0.320)	p < 0.001
90-day mortality	HR = 0.242 (0.159–0.367)	*p* < 0.001	HR = 0.240 (0.158–0.365)	p < 0.001	HR = 0.230 (0.150–0.352)	p < 0.001

Model 1: unadjusted.

Model 2: adjusted for age; gender; BMI.

Model 3: adjusted for age; gender; BMI; APSIII; SAPSII; metformin; OASIS; CRRT; PLT; CHARLSON; creatine; VP; glucose; PH; hemoglobin; calcium; RBC; HCT; SOFA; PCO2; bilirubin; AG; BUN; chloride; TCO2; lactate.

### Subgroup analysis

**FIGURE 4:**
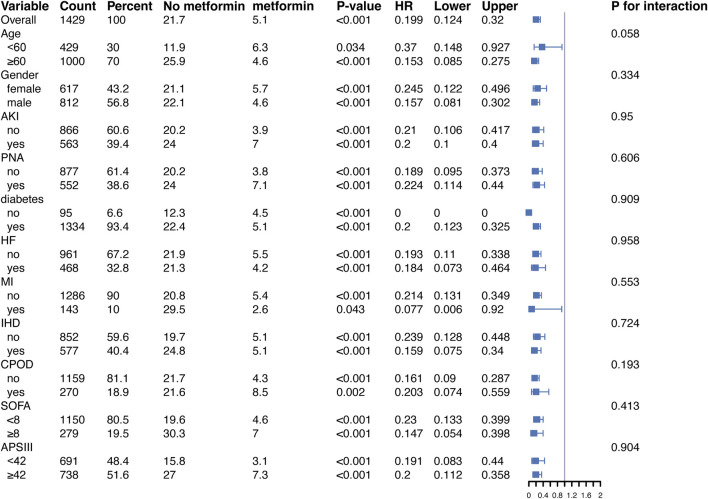
Protective effects of metformin on 30-day all-cause mortality across subgroups in the fully adjusted Cox regression model. Abbreviations: HR, hazard ratio; AKI, acute kidney injury; PNA, pneumonia; HF, heart failure; MI, myocardial infarction; IHD, ischemic heart disease; COPD, chronic obstructive pulmonary disease; SOFA, sequential organ failure assessment; APSIII, acute physiology score III.

In addition, to validate the relationship between metformin and 30-day all-cause mortality in patients with ARF, we conducted subgroup analyses according to age, gender, BMI, SOFA score, APSIII score, and diabetes status. Figure 4, in the fully adjusted model, metformin demonstrated a significant protective effect in multiple groups of patients. For example, there was a significant association between metformin and 30-day all-cause mortality in both male (HR = 0.157, 95% CI 0.081–0.302) and female (HR = 0.245, 95% CI 0.122–0.496) patients, as well as for patients diagnosed with PNA (HR = 0.224, 95% CI 0.114–0.440) and those without PNA (HR = 0.189, 95% CI 0.095–0.373), the protective effect of metformin remained unchanged.

**FIGURE 5:**
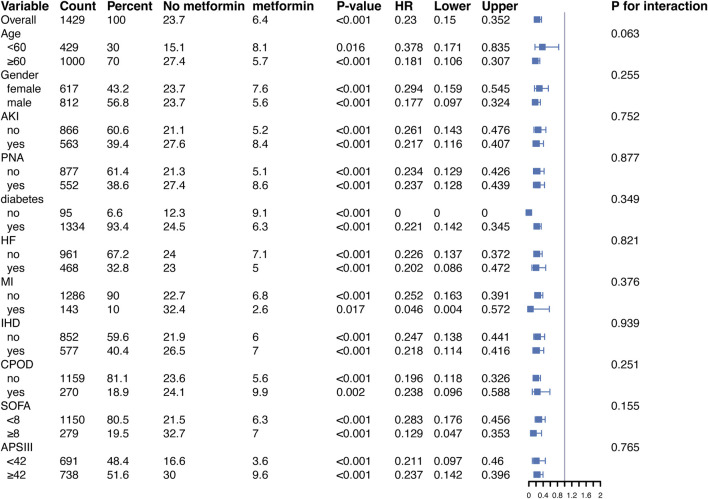
Protective effects of metformin on 90-day all-cause mortality across subgroups in the fully adjusted Cox regression model. Abbreviations: HR, hazard ratio; AKI, acute kidney injury; PNA, pneumonia; HF, heart failure; MI, myocardial infarction; IHD, ischemic heart disease; COPD, chronic obstructive pulmonary disease; SOFA, sequential organ failure assessment; APSIII, acute physiology score III.

Of note, in diabetic patients, metformin significantly reduced the risk of death (HR = 0.201, 95% CI 0.123–0.326, p < 0.001). In contrast, in the non-diabetic subgroup, the reference group (non-metformin users) had a small sample size (95 patients) and a low number of events (10 deaths), leading to instability in the Cox regression model. This may explain the computational limitations and the unexpected HR of 0 observed for this group. Despite these issues, the protective effect of metformin was directionally consistent across subgroups, suggesting potential benefits even in non-diabetic patients. Moreover, similar trends were observed in the stratified analysis of 90-day all-cause mortality (Figure 5).

### Sensitivity analysis

**FIGURE 6:**
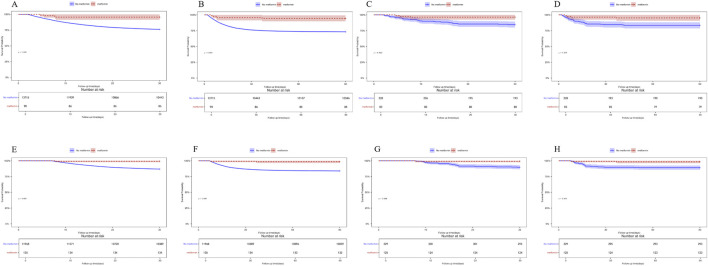
**(A)** 30-day mortality in the unmatched cohort, landmark time at 48 h **(B)** 90-day mortality in the unmatched cohort, landmark time at 48 h **(C)** 30-day mortality in the PSM cohort, landmark time at 48 h **(D)** 90-day mortality in the PSM cohort, landmark time at 48 h **(E)** 30-day mortality in the unmatched cohort, landmark time at 7 days. **(F)** 90-day mortality in the unmatched cohort, landmark time at 7 days. **(G)** 30-day mortality in the PSM cohort, landmark time at 7 days. **(H)** 90-day mortality in the PSM cohort, landmark time at 7 days.

In the landmark analyses at 48 h and 7 days after ICU admission, Kaplan–Meier curves showed that metformin use within each respective window was associated with significantly lower 30-day and 90-day mortality compared with non-use (log-rank p < 0.05 for all comparisons). These associations were consistent in both the unmatched and PSM cohorts (Figure 6). The concordant findings across two landmark time points strengthen the robustness of our results and further reduce concerns about immortal time bias.

## Discussion

This study examined the relationship between metformin use and mortality in patients with ARF and showed that metformin use significantly reduced in-hospital and ICU mortality. These findings support that metformin may play a significant role in the holistic treatment of ARF.

ARF is a prevalent cause of hospitalization and admissions to critical care units, resulting from many etiologies. According to previous studies, pre-discharge mortality is as high as 40% for patients with ARF requiring ICU admission and invasive mechanical ventilation (Azoulay et al., 2019). Early recognition and treatment of ARF not only helps to reduce complications but also reduces ICU and hospitalization time and significantly improves survival.

Lung inflammation is a central mechanism in the pathogenesis of ARF, often triggered by microbial infections such as pneumonia or acute exacerbations of COPD, and may progress to acute respiratory distress syndrome (ARDS), a severe form of diffuse inflammatory lung injury (Umakanthan et al., 2020; Hashimoto et al., 2017; Thompson et al., 2017). These conditions are characterized by dysregulated immune responses and excessive release of pro-inflammatory cytokines, leading to alveolar damage and impaired gas exchange (Parsons et al., 2005; Wedzicha and Seemungal, 2007; Ferguson et al., 2012). Metformin, a widely used antidiabetic agent, has attracted growing interest for its pleiotropic effects beyond glycemic control. Mechanistically, it activates AMPK, suppresses oxidative stress, and inhibits the release of pro-inflammatory cytokines (Kristófi and Eriksson, 2021; Xian et al., 2021). These immunomodulatory properties have been supported by clinical studies in populations with chronic inflammatory conditions such as type 2 diabetes and polycystic ovary syndrome, with reductions in markers like C-reactive protein (CRP) (Haffner et al., 2005; Lund et al., 2008; Tsilchorozidou et al., 2009; Diamanti-Kandarakis et al., 2006). Such effects provide a biological rationale for exploring metformin as adjunctive therapy in critically ill patients with inflammatory syndromes such as ARF.

The COVID-19 pandemic has further drawn attention to the immunomodulatory potential of metformin. Several retrospective studies and early meta-analyses suggested that metformin use in patients with type 2 diabetes was associated with reduced mortality and severity in COVID-19 (Bramante et al., 2021; Khunti et al., 2021; Lalau et al., 2021; Li et al., 2021; Lukito et al., 2020). However, recent evidence from randomized controlled trials offers a more nuanced perspective. A comprehensive systematic review and meta-analysis of RCTs concluded that metformin did not significantly reduce all-cause mortality or risk of clinical deterioration in hospitalized COVID-19 patients (Chowdhury et al., 2025). These findings suggest that while metformin’s theoretical mechanisms of action remain plausible, its clinical efficacy in acute viral ARDS, such as COVID-19-related ARF, is still inconclusive. The conflicting results underscore the complexity of metformin’s effects in acute illness. Differences in study populations (e.g., diabetes status), timing of drug exposure, and underlying ARF etiology (e.g., viral vs non-viral) may contribute to the variability in findings across studies (Bramante et al., 2021). Our study complements this body of work by focusing on a broader ARF population in the ICU setting. Nevertheless, the lack of benefit observed in recent meta-analyses of COVID-19 RCTs highlights the need for cautious interpretation of observational findings.

In the present study, metformin use significantly reduced in-hospital mortality and ICU mortality in ARF patients in fully adjusted multivariate models. These results suggest that metformin may not only be protective against death associated with systemic inflammation and metabolic disorders, but may also have potential benefits for the complex clinical state of ICU patients (Zhou et al., 2001). Notably, patients with ARF often die due to MODS or severe lung injury (Phua et al., 2009; Bellani et al., 2016; Herridge et al., 2003), whereas metformin may attenuate multiple organ dysfunction by suppressing inflammatory responses and oxidative stress (Cameron et al., 2016; Isoda et al., 2006). These findings further suggest that metformin may have broad clinical potential in the comprehensive treatment of critically ill patients.

To further assess the robustness of our findings against potential unmeasured confounding, we calculated the E-value for the association between metformin use and in-hospital mortality. The observed odds ratio of 0.202 corresponds to an E-value of 9.37 (Supplementary Figure S2), which represents the minimum strength of association that an unmeasured confounder would need to have with both metformin use and mortality—beyond the measured covariates—to fully explain away the observed association. This high E-value suggests that residual confounding is unlikely to entirely account for the magnitude of the observed protective effect, thus reinforcing the robustness of our findings under plausible assumptions (VanderWeele and Ding, 2017).

While our findings suggest a potential benefit of metformin in ICU patients with ARF, several limitations should be acknowledged. First, this was a retrospective observational study. Although PSM balanced baseline characteristics, we cannot completely rule out the effect of residual confounding (e.g., the patient’s current and past medical history) on outcomes. In particular, the “non-user” group may include patients who were not prescribed metformin due to contraindications, introducing potential reverse causality and overestimating the protective effect. Moreover, PSM and standard regression models primarily adjust for baseline variables and cannot fully account for time-varying confounding. Future studies should consider advanced methods such as marginal structural models to address these dynamic treatment effects. Second, ARF was identified using ICD codes, which may not fully capture the clinical diagnosis. Some patients may have been misclassified due to transient hypoxia or rule-out diagnoses, while others with true ARF may have been missed if classified under specific conditions (e.g., severe pneumonia or cardiogenic pulmonary edema). Moreover, the study population was derived from a single academic ICU and comprised mostly critically ill patients. These factors may limit the generalizability of our findings to less severe or non-ICU ARF populations. Given the clinical heterogeneity of ARF in etiology and severity, treatment effects of metformin may vary across subgroups. Future studies should explore whether specific ARF subphenotypes derive greater benefit (Yang et al., 2024). Finally, the associations observed in this study are correlational rather than causal. Although we applied robust statistical adjustments, causal inference remains limited. Future prospective or target trial emulation studies are needed to better estimate the effect of metformin in this setting (Yang et al., 2025; Hernán et al., 2025).

## Conclusion

In this retrospective cohort study, metformin use was associated with reduced in-hospital, ICU, 30-day, and 90-day mortality among ICU patients with ARF. These findings suggest potential benefits of metformin beyond glycemic control. However, due to the observational nature of the study and possible residual confounding, the results should be interpreted with caution. Further prospective studies are needed to confirm these associations and clarify their clinical relevance.

## Data Availability

The raw data supporting the conclusions of this article will be made available by the authors, without undue reservation.
