# Supplementary figures and images for: Correlation between metformin use and mortality in acute respiratory failure: a retrospective ICU cohort study
Source: Front Pharmacol. 2025 Aug 26;16:1584230. doi: 10.3389/fphar.2025.1584230 (PMC12417518; doi:10.3389/fphar.2025.1584230)

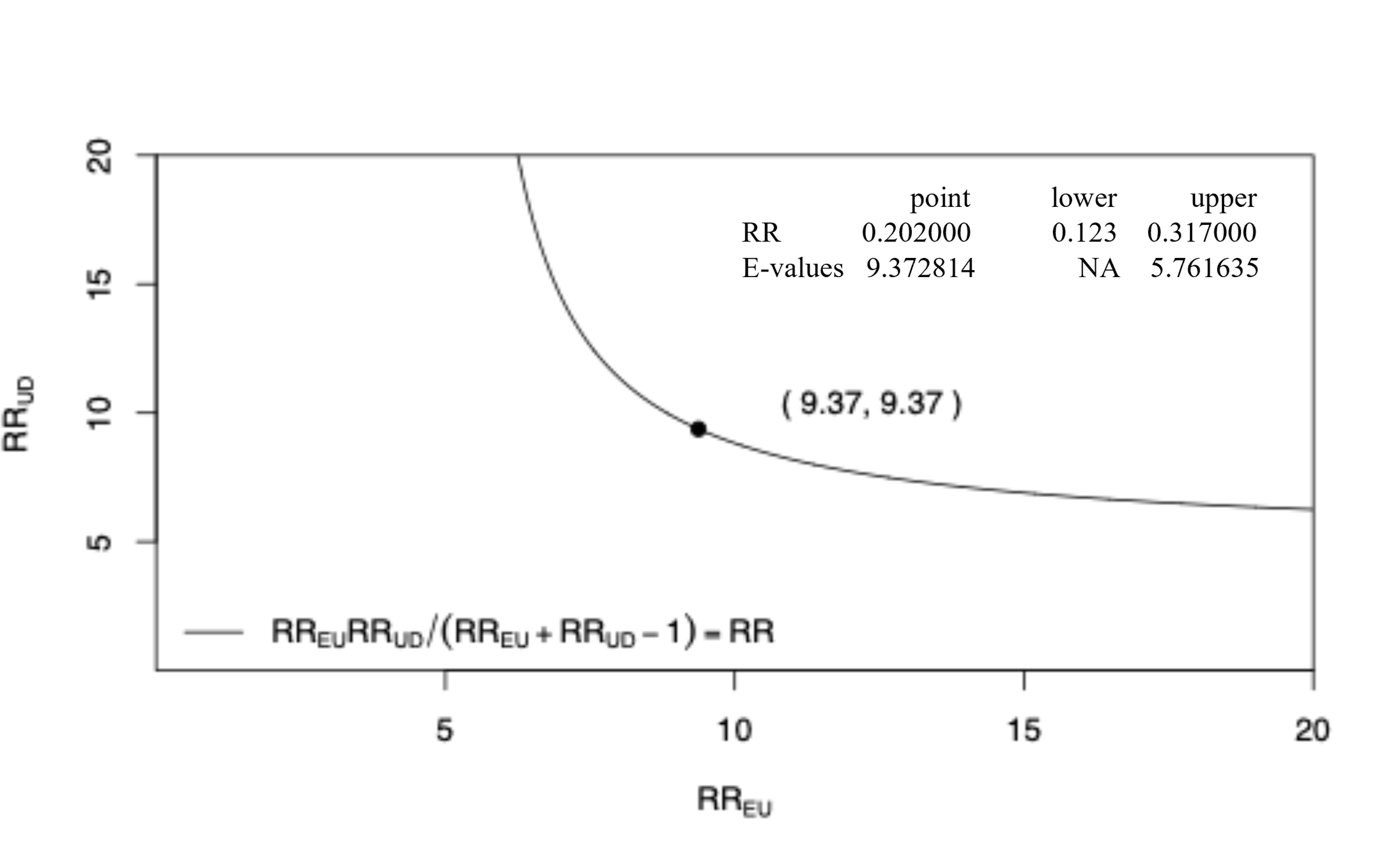

Supplement: Supplementary file 1 [file Image2.tif]

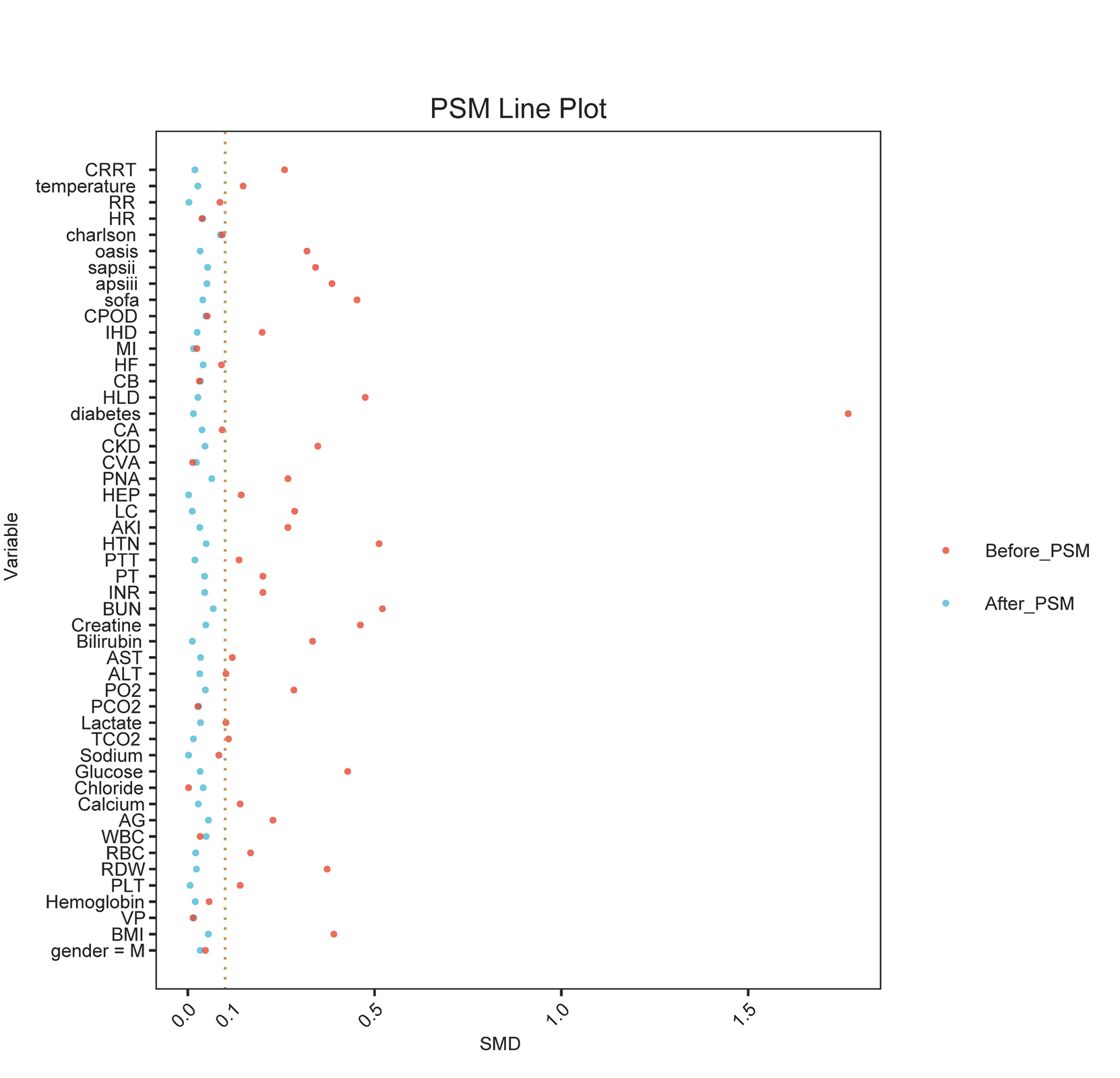

Supplement: Supplementary file 2 [file Image1.tif]
